# Research advances in brain-computer interfaces in oncology: from cancer pain management to neuroengineering for brain tumors and tumor-neural-immune interactions

**DOI:** 10.3389/fimmu.2026.1953098

**Published:** 2026-10-21

**Authors:** Yawen Song, HongXing Guo, Zhen Yu, Aihua Wang, Jiaxin Li, Zhengcun Pei, Jiajing Chen, Xinyun Zhang, Hongsheng Guo, Xue Wu, Jingkun Zhang, Chenxuan Wu, Ximo Wang

**Affiliations:** 1 Central Hospital, Tianjin University, Tianjin, China; 2 Tianjin Key Laboratory of Extracorporeal Life Support for Critical Diseases, Tianjin, China; 3 Tianjin Artificial Cell Engineering Technology Research Center, Tianjin, China; 4 Tianjin Institute of Hepatobiliary Disease, Tianjin, China; 5 Tianjin Institute of Geriatrics, Tianjin Third Central Hospital Branch, Tianjin, China; 6 Department of Oncology, Central Hospital, Tianjin University, Tianjin, China; 7 Department of Laboratory, Central Hospital, Tianjin University, Tianjin, China; 8 Department of General Surgery, Central Hospital, Tianjin University, Tianjin, China; 9 Medical School, Tianjin University, Tianjin, China; 10 Institute for Global Health Sciences, University of California, San Francisco, San Francisco, CA, United States; 11 Department of Physiology, University of California, San Francisco, San Francisco, CA, United States

**Keywords:** brain tumor, brain-computer interface, cancer pain, electrocorticography, tumor-nerve-immune axis

## Abstract

Brain-computer interfaces (BCIs) have made significant progress in fields such as neurorehabilitation, motor reconstruction, and alternative communication by capturing, decoding, and providing feedback on brain activity. In contrast, the application of BCI in oncology has a relatively late start, but its development path has gradually become clearer in recent years. On the one hand, brain-signal interfaces based on neural feedback and closed-loop regulation have begun to be used for interventions targeting cancer-related neurological symptoms, particularly cancer pain and chemotherapy-induced peripheral neuropathy (CIPN), suggesting that supportive cancer care is shifting from empirical symptomatic management toward quantifiable, feedback-based, and adjustable neural state management. On the other hand, in the field of brain tumors, ECoG recording, high-density neural recording, and real-time decoding are providing complementary information on abnormal peritumoral networks and tumor-associated epilepsy within established function-guided surgical workflows. These recording and decision-support approaches are related to BCI engineering but are not automatically BCIs or substitutes for direct electrical stimulation mapping. More importantly, advances in tumor neuroscience suggest that neural activity not only reflects the disruption of host brain tissue by the tumor but may also participate in glioma growth and invasion through circuit remodeling and microenvironmental changes. This article provides a review of the fundamentals of BCI technology, the management of cancer-related symptoms, brain-computer interfaces in brain tumors, tumor neuroscience, tumor-neural-immune interactions, and their translational challenges. Current evidence suggests that the significance of BCI in oncology is no longer limited to assistive devices but is evolving into a novel engineering platform that integrates symptom control, surgical decision-making, and research into disease mechanisms.

## 1 Introduction

Cancer remains one of the leading causes of disease burden and mortality worldwide. According to GLOBOCAN 2022 data, there were approximately 20 million new cancer cases and 9.7 million cancer-related deaths globally in 2022; against the backdrop of future population growth and aging, the number of new cancer cases worldwide is projected to exceed 35 million annually by 2050 (1). As survival times for cancer patients increase, the significance of cancer-related symptoms and treatment-related sequelae continues to rise. For many patients, the burden of the disease extends beyond the tumor itself; neurological issues such as cancer pain (2–6), chemotherapy-induced peripheral neuropathy (CIPN) (7–12), and brain tumor-related epilepsy and functional impairment (13–18) can substantially affect quality of life and treatment tolerance. BCI studies in predominantly non-oncological populations with severe neurological disability provide a technological basis for exploring communication and motor support in selected oncology settings (19–24). Contemporary epidemiological updates and guideline syntheses reinforce that symptom burden, pain prevalence, and neurological toxicities remain major determinants of survivorship outcomes, even when tumor-directed treatments are successful (4–8).

Among these, cancer pain represents one of the most significant unmet needs in supportive cancer care. Cancer-related pain is a complex condition driven by a combination of the tumor itself, treatment-induced damage, and psychosocial factors (25, 26). Although the World Health Organization and several international societies have proposed systematic strategies for cancer pain management (27–29), a significant proportion of patients still experience inadequate pain relief in clinical practice, while the side effects, tolerance, and limitations of long-term use of opioid medications continue to influence treatment decisions (30). This reality has driven the development of neuromodulation, non-pharmacological interventions, and multimodal pain management approaches. Recent NCCN and ASCO guidance further supports structured pain assessment, opioid stewardship, and multimodal approaches that include non-pharmacological and rehabilitative strategies when appropriate (5, 6, 29, 31, 32).

Brain-computer interfaces were initially defined as a technology that bypasses traditional neuromuscular output pathways to directly exchange information with external systems using brain activity (33–35). Over the past two decades, with advancements in invasive recording, high-density arrays, flexible electrodes, neural decoding algorithms, and artificial intelligence methods, BCIs have gradually progressed from proof-of-concept to clinical translation, demonstrating unprecedented capabilities in restoring communication for patients with severe paralysis, converting brain signals into text, and developing neural prosthetics for speech generation (19–24). In contrast, oncology was not one of the earliest application areas for BCIs, but this situation is changing. Recent BCI and neuroprosthetic studies have also strengthened the translational rationale for adaptive, high-performance, and clinically interpretable neural interfaces (36–42).

The application of BCI in oncology stems primarily from the widespread need to manage neurological symptoms in cancer patients. Chronic symptoms, such as cancer pain and CIPN, are often accompanied by significant changes in central nervous system plasticity. Opioids remain important for moderate-to-severe cancer pain, but adverse effects require monitoring and individualized prescribing (6, 29, 30, 32). Treatment of established painful CIPN remains limited and should follow condition-specific guidance (7, 8). Second, in the field of brain tumors, the relationship between the tumor and brain networks is far more complex than a simple anatomical mass. Issues such as peritumoral abnormal discharges, functional reorganization, tumor-associated epilepsy, and the protection of language and motor areas require surgeons not only to identify lesion boundaries but also to understand the state of the network in which the tumor is embedded (13–16, 46–48). Furthermore, advances in tumor neuroscience suggest that neural activity is not merely a consequence of tumor symptoms but may also directly contribute to glioma growth, invasion, and circuit remodeling (49–55). Accordingly, this review considers conventional BCIs alongside related brain-signal interfaces centered on signal acquisition, decoding, feedback, and modulation. This broader conceptual scope does not classify routine ECoG recording or direct electrical stimulation mapping as BCIs. This expansion is supported by converging evidence from CIPN guidelines and neurofeedback trials (7, 56–59), intraoperative ECoG/peritumoral network studies (13–16, 46, 47), functional mapping studies and reviews (48, 62–64), and recent tumor-neuroscience (65–68) and neuroimmune-oncology work (69–73) (**Figure 1**).

**Figure 1:**
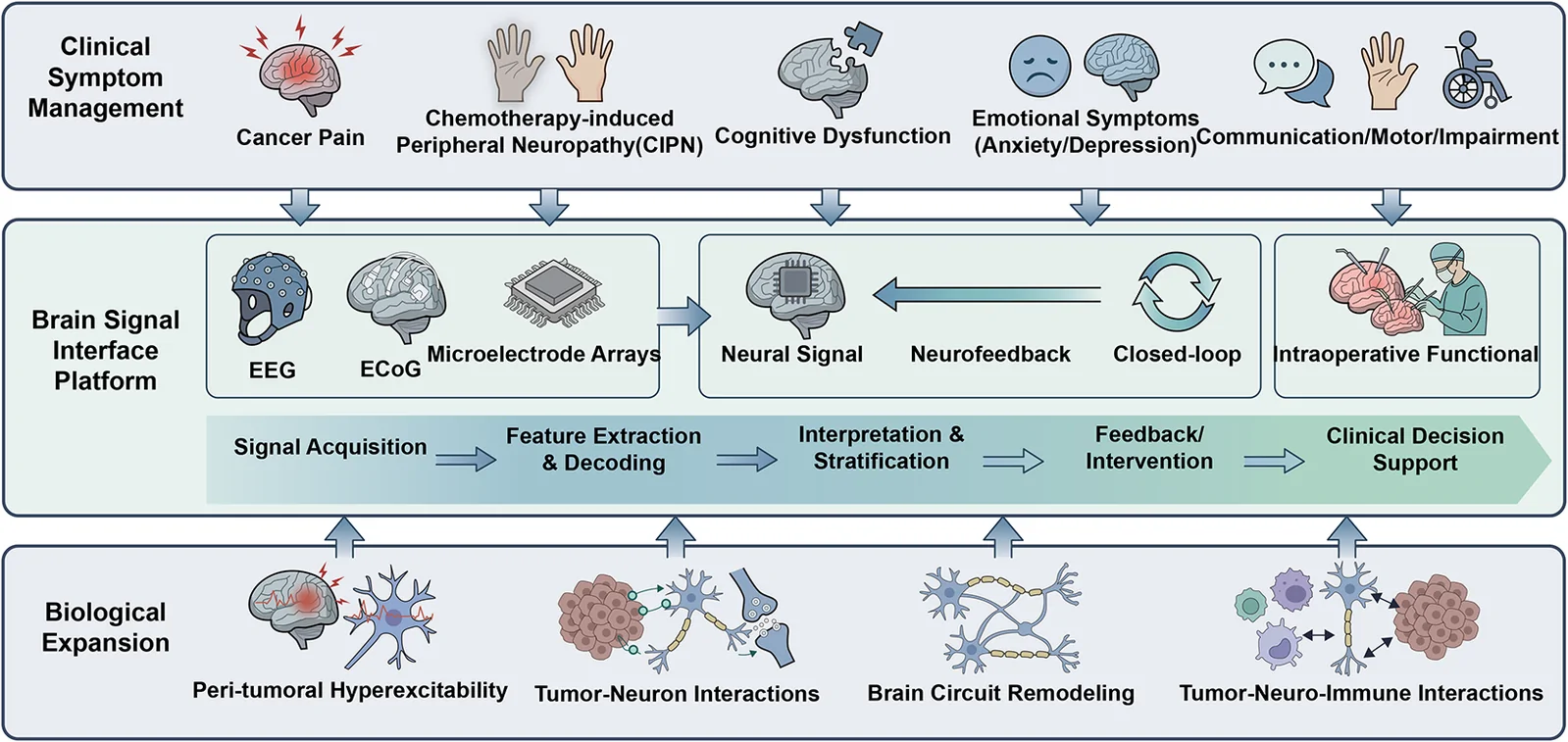
An overarching conceptual framework for brain-computer interfaces in oncology. The upper layer represents clinical symptom management, encompassing cancer pain, chemotherapy-induced peripheral neuropathy, cognitive impairment, emotional symptoms, and impaired communication/motor function; the middle layer represents the brain signal interface/neuroengineering platform, encompassing EEG, ECoG, microelectrode arrays, neural decoding, neural feedback, closed-loop control, and intraoperative functional mapping; the lower layer is the biological extension layer, encompassing peritumoral hyperexcitability, tumor-neuron interactions, brain circuit remodeling, and tumor-neuron-immune interactions. The central technological axis is “signal acquisition-feature extraction and decoding-interpretation and stratification-feedback/intervention-clinical decision support.” This is an expanded conceptual framework that includes conventional BCIs and related neuroengineering tools, rather than an established classification of all depicted methods as BCIs. Intraoperative functional mapping includes cortical and subcortical direct electrical stimulation (DES); ECoG recording provides complementary information and does not independently establish functional boundaries.

Based on this, this paper addresses five key questions: First, what are the developments and fundamental forms of BCI technology? Second, why has BCI been investigated first in the management of cancer-related symptoms, such as cancer pain? Third, how can BCI-related recording and decision-support approaches complement established functional preservation and resection strategies in brain tumors? Fourth, how do tumor neuroscience and tumor-neural-immune interactions provide biological context for future BCI research in oncology? Fifth, what challenges remain in evidence generation, technology integration, and ethical regulation? This review frames BCI in oncology not merely as an assistive technology, but as an engineering platform for neural-state measurement, adaptive feedback, intraoperative decision support, and mechanism-oriented neuro-oncology research.

Literature identification and selection. This article is a narrative review, not a systematic review or meta-analysis. To improve transparency during revision, targeted supplementary searches were conducted on 19 JUNE 2026 in Europe PMC, including its PubMed/MEDLINE records, with verification against PubMed records and publisher pages where available. Searches covered publications dated from 1900 to 19 JUNE 2026 and combined title/abstract terms for “brain-computer interface” or “neurofeedback” with “cancer,” “oncology,” or “chemotherapy-induced peripheral neuropathy”; “electrocorticography,” “direct electrical stimulation,” or “functional mapping” with “glioma” or “brain tumor”; and BCI terms with “definition,” “passive,” “user-centered,” “clinical benefit,” or “safety.” A complementary search combined “cancer neuroscience,” “tumor neural immune,” or “neuro-immune” with cancer, glioma, or pancreatic terms. Reference lists of relevant sources were also consulted. English-language clinical studies, engineering studies, mechanistic studies, reviews, guidelines, and relevant ethical analyses were selected for direct relevance to the claims discussed, prioritizing oncology-specific evidence and using non-oncological work as explicitly identified technical or conceptual context. Peripheral tumor-neural-immune studies were included as mechanistic background, not evidence of BCI efficacy. Duplicate reports, unrelated articles, and unsupported extrapolations were excluded from the relevant evidence claims. Selection was purposive rather than exhaustive; no prospective protocol or formal risk-of-bias assessment was used, and publication and selection bias remain possible. This description documents the revision-stage search and does not imply a prospectively recorded search protocol for the original draft.

## 2 An overview of brain-computer interface technology

A brain-computer interface (BCI) is a system that establishes a direct information pathway between the brain and external devices, enabling the brain to output information, control devices, or regulate feedback without relying on peripheral nerves or muscles (33–35). Its basic process typically consists of four stages: brain signal acquisition, signal preprocessing and feature extraction, command translation, and output feedback (33–35). Signal sources may include electroencephalography (EEG), electrocorticography (ECoG), intracortical microelectrodes, and functional imaging; different acquisition methods involve trade-offs in terms of spatio-temporal resolution, invasiveness, and clinical accessibility (23, 33–35). Clinical BCI evaluation should address patient benefit (74), technical reliability (75), user-centered usability (79), and adverse events during longitudinal follow-up (81), rather than decoding accuracy alone (**Figure 1**).

Based on the degree of invasiveness, BCIs can be classified into invasive, semi-invasive, and non-invasive systems. Invasive systems offer higher signal quality and support more complex decoding and higher-precision control, but they also pose risks associated with surgery, biocompatibility, and long-term stability; non-invasive systems, such as EEG, carry lower risks and offer greater accessibility, making them more suitable for neurofeedback training and large-scale implementation; however, they have limitations in terms of spatial resolution and signal-to-noise ratio (23, 33–35). Based on information flow, BCIs can be further classified into unidirectional systems and closed-loop bidirectional systems. The latter can both record brain states and implement real-time feedback or modulation based on decoding results, making them more aligned with the future development direction of precise neuromodulation (34, 76). This closed-loop orientation is particularly relevant for oncology because symptom states and tumor-associated network excitability can vary over time, as illustrated by neurofeedback evidence in chronic neuropathic symptoms (56–59, 76–78) and electrophysiological studies of tumor-related networks (13–16, 60, 61).

Currently, the most mature clinical applications of BCI remain concentrated in neuromotor rehabilitation and assisted communication. Invasive BCI has enabled high-performance spelling, brain-to-text conversion, speech decoding, and neural prosthesis control in patients with severe paralysis (19–24). These technological advancements have laid the methodological foundation for the integration of BCI into oncology. For cancer patients, the significance lies not only in functional compensation but also in providing a new pathway for quantifying neural states and intervening, thereby gradually bringing symptom management, intraoperative decision-making, and disease progression monitoring under a single technological framework. Recent speech and communication neuroprostheses demonstrate high-throughput decoding and rapid calibration in selected participants with paralysis; these are engineering benchmarks rather than established oncology outcomes (39–42) (**Table 1**).

**Table 1:** BCI modalities and related neuroengineering applications in oncology.

Interface modality	Oncology-facing use case	Engineering value	Principal limitations	Representative evidence
EEG neurofeedback/non-invasive BCI	Cancer pain and chronic CIPN symptom management; longitudinal neural-state monitoring.	Low surgical risk; scalable; compatible with rehabilitation and home-based digital care.	Lower spatial resolution; placebo/sham-control sensitivity; training burden and inter-individual variability.	CIPN RCT 56; broader pain review/study evidence 57-59; neurofeedback framework 76.
ECoG/intraoperative cortical recording	Brain-tumor surgery, tumor-related epilepsy, peritumoral hyperexcitability, and task-based language assessment.	Records cortical activity for intraoperative decision support; may complement independent DES mapping.	Requires operative exposure; coverage varies; abnormal or task-related activity alone does not establish functional necessity or safe resection limits.	ECoG/HFO studies 13-16,60,61; recording/mapping studies 46,47; CCEP review 48.
Intracortical microelectrode arrays	High-performance communication and potential neuroprosthetic support for patients with severe tumor- or treatment-related deficits.	High signal fidelity and high-throughput decoding; establishes benchmarks for future oncology-facing neural interfaces.	Invasive implantation; long-term stability, infection risk, calibration burden, and maintenance remain major barriers.	Communication studies 19,20,39,41; engineering review 75; rapid calibration 80; longitudinal safety 81.
Flexible/high-density cortical arrays	Fine-grained cortical recording and task-related functional inference alongside established mapping during tumor resection.	Improved conformability and spatial sampling; may reduce stimulation burden in selected validated paradigms.	Early evidence; recording-based inference does not establish functional necessity; outcome validation and workflow integration are required.	High-density cortical recording 46; task-related inference 63; array engineering 109,110. Ref. 109 is preclinical.
Closed-loop neuromodulation platforms	Adaptive symptom control, perioperative risk monitoring, and future mechanism-guided intervention studies.	Transforms BCI from readout to feedback and control; supports patient-specific adaptation over time.	Requires robust biomarkers, safety thresholds, explainable algorithms, and prospective clinical validation.	Closed-loop/neurofeedback rationale (56, 74–76)
Multimodal AI-enabled neural interface	Integration of neural signals with imaging, pathology, molecular data, immune profiling, and patient-reported outcomes.	Proposed integration of symptoms, circuits, and biological measurements for prospective neuro-oncology research.	Data drift, bias, interoperability, privacy, and clinical interpretability remain unresolved implementation issues.	Clinical benefit 74; robustness 75; usability 79; safety 81; ethics and governance 82-92.

BCI, brain-computer interface; CIPN, chemotherapy-induced peripheral neuropathy; DES, direct electrical stimulation; ECoG, electrocorticography; EEG, electroencephalography; HFO, high-frequency oscillation; RCT, randomized controlled trial.

## 3 Management of cancer-related symptoms

Cancer pain is one of the most common and debilitating symptoms experienced by cancer patients. The International Classification of Pain has established a distinct framework for chronic cancer-related pain, emphasizing its complex nature, which is driven by a combination of the tumor itself, treatment-related damage, and psychosocial factors (25, 26). From an epidemiological and clinical outcomes perspective, cancer pain is associated with substantial suffering, impaired daily function, and reduced quality of life (2, 3, 27). Persistent pain is therefore not merely a symptom but also a critical phenotypic endpoint reflecting the quality of care throughout the cancer journey. More recent prevalence syntheses and cancer-pain guidelines confirm that pain remains a high-burden, undertreated syndrome across active treatment and survivorship settings (4–6).

A key reason for the intractability of cancer pain lies in its pathophysiological basis, which is characterized by multiple drivers and converging pathways. Tumor compression or infiltration of bones and nerves, the release of inflammatory mediators, and treatment-related neurotoxicity collectively constitute a continuous input of noxious stimuli (93–96); simultaneously, central sensitization and neuroinflammatory processes cause pain to gradually evolve into a plastic pathological state at the neurological level (43–45, 97). This is particularly evident in CIPN, where some patients continue to experience pain, numbness, and sensory abnormalities even years after discontinuing medication, suggesting that the pain network has undergone long-term remodeling (7–12). Current CIPN literature further supports a combined peripheral-central model in which axonal injury (9, 11, 12), neuroinflammation (44, 45, 97), cortical reorganization (10), and persistent sensory dysfunction contribute to chronic symptoms (7, 8). Exercise and rehabilitation studies report symptom and functional benefits (12, 98–101), but do not by themselves establish a central neuroplastic mechanism or BCI efficacy.

Although the WHO, ESMO, ASCO, and guidelines from various countries have proposed relatively systematic strategies for cancer pain management (27–29), including drug stratification, adjunctive therapies, and integrative medicine interventions, inadequate pain relief remains common in clinical practice. Although opioids form a crucial foundation for the management of moderate-to-severe cancer pain, limitations related to their side effects, tolerance, and long-term use persist (27–30). Consequently, multimodal analgesia, neuromodulation, and interventions with low systemic burden have gradually gained prominence, and it is against this backdrop that BCI has emerged as a novel approach in supportive cancer care.

The core rationale for using BCI to treat cancer pain lies in the fact that pain is not determined solely by peripheral sensory input, but rather is a subjective experience integrated by the sensory cortex, limbic system, and descending modulatory networks (43, 44, 76). If neurofeedback training can enable patients to modulate pain-related brain activity patterns, it may reshape pain perception and promote neural regulatory processes associated with endogenous analgesia (56, 76). A key oncology-specific study is the randomized, double-blind, placebo-controlled trial by Prinsloo et al. in breast cancer survivors with chronic CIPN. After 20 sessions, both targeted BCI neurofeedback and placebo feedback groups reported symptom improvement relative to a waitlist; continued improvement at one month was observed only in the targeted BCI group (56). These findings support further study of durability and specific neurofeedback effects, rather than an unqualified claim of superiority over placebo or efficacy for all cancer pain. The significance of this study lies not only in proposing a potential new therapy but also in suggesting that supportive cancer care may be entering a “neuro-engineered” phase, wherein brain activity is no longer merely a passive indicator of disease outcomes but may become an active component within the therapeutic loop. A scoping review and a systematic review/meta-analysis address chronic pain more broadly (57, 58), while a separate clinical study evaluated peripheral neuropathic pain (59); these are not all cancer-specific studies. Longitudinal CIPN data (77) and a home-based neuropathic-pain pilot (78) further motivate larger sham-controlled studies and mechanistic endpoints.

The potential advantages of BCI in the context of cancer pain are primarily reflected in three aspects: First, it offers a non-pharmacological, personalized, and trainable intervention pathway suitable for long-term management; second, it extends symptom assessment from subjective scales to dynamic brain signal indicators, adding a new dimension to efficacy evaluation and mechanism research; third, its closed-loop nature theoretically enables the potential to adjust feedback strategies in real time based on neural states (56, 76). However, current evidence remains limited, with generally small sample sizes, significant heterogeneity in training protocols, and some results that cannot fully rule out the placebo effect. Therefore, a more prudent approach is to view BCI as a potential adjunctive strategy for cancer pain management rather than a replacement for current standard treatments. Accordingly, neurofeedback-based BCI should be interpreted as an evidence-generating adjunct rather than a stand-alone replacement for guideline-directed analgesia (57–59, 77, 78).

In addition to cancer pain, BCI-related communication and rehabilitation technologies may provide methodological inspiration for selected cancer-related neurological sequelae, although direct oncology-specific evidence remains limited (36–42). Exercise and rehabilitation are being studied for CIPN and may improve symptoms and function (12, 98–101). Their integration with BCI-supported care is a proposed research direction, not an outcome established by these studies.

## 4 Brain-computer interfaces in brain tumors: from surgical resection to networked neuroengineering

If cancer pain represents BCI’s peripheral entry point into oncology, then brain tumors constitute its most natural and structurally significant application scenario. Brain tumor surgery has always faced a fundamental tension: maximizing tumor resection while preserving neural function as much as possible. Structural imaging defines radiological lesion extent and anatomical relationships, whereas functional imaging provides indirect estimates of task-related activation or connectivity. Direct electrical stimulation (DES) of cortical and subcortical structures tests function intraoperatively and identifies functional constraints on resection; it is not a method for defining static anatomical tumor boundaries (62–64, 102). Function-guided resection, particularly during awake glioma surgery, is an established principle: in selected patients, resection may extend beyond MRI-visible tumor margins but must respect functionally critical cortical and subcortical structures (102, 106, 107). Contemporary electrophysiological recording can add dynamic information about cortical activity and network excitability to this established framework (13–16, 46, 47).

Four distinct types of boundary must therefore be distinguished. Oncological or tumor-infiltration boundaries refer to the distribution of neoplastic cells, which in diffuse glioma may extend beyond MRI-visible abnormalities and may not form a sharply demarcated edge. Anatomical/radiological boundaries describe structural landmarks and imaging-defined lesion extent. Functional or eloquent boundaries identify cortical and subcortical structures whose disruption risks neurological impairment, assessed through patient-specific functional mapping. Epileptogenic/electrophysiological boundaries describe seizure-related networks or the spatial distribution of recorded abnormalities; these terms are themselves not interchangeable, because interictal discharges or HFOs alone do not establish the full epileptogenic zone. These distributions may overlap but need not coincide. Electrically abnormal tissue may be functionally expendable, functionally critical, or partly overlap eloquent cortex; its resectability requires an independent functional assessment (13, 14, 16, 60–64, 102, 106) (**Figure 2**).

**Figure 2:**
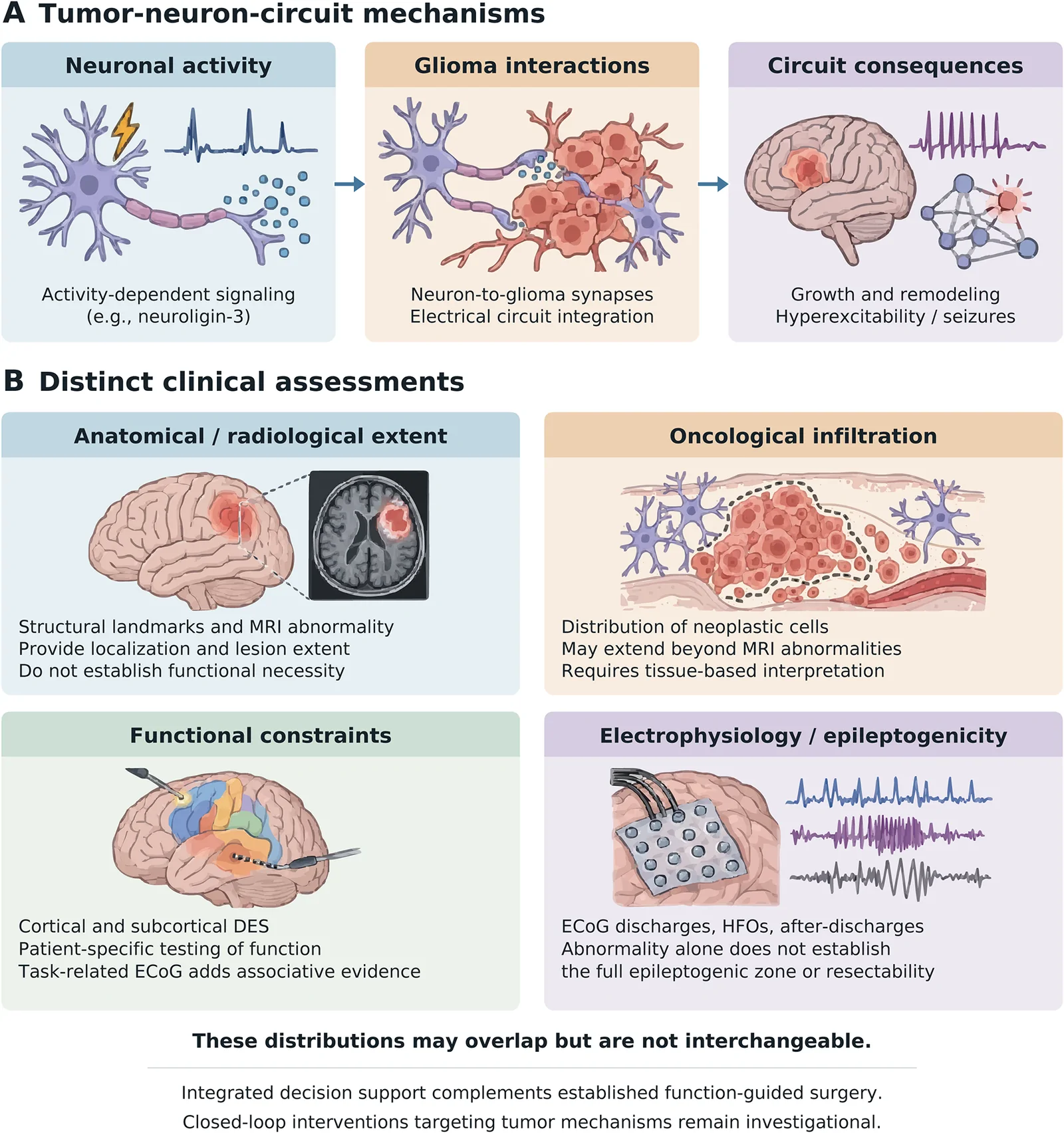
Tumor–neuron–circuit mechanisms and complementary clinical assessments in glioma. **(A)** Activity-dependent signaling, neuron-to-glioma synaptic input, and electrical circuit integration are associated with glioma growth and circuit remodeling, alongside hyperexcitability and seizures. **(B)** Anatomical/radiological extent, oncological infiltration, functional constraints, and electrophysiological abnormalities represent distinct assessments whose distributions may overlap but are not interchangeable. ECoG records cortical activity and can provide associative evidence during task-related testing; functional constraints require patient-specific assessment, including cortical and subcortical direct electrical stimulation (DES). Neither abnormal ECoG activity nor a task-related ECoG response alone establishes resectability. Integrated decision support complements established function-guided surgery, while closed-loop interventions targeting tumor-related mechanisms remain investigational.

Molecular classification addresses a separate clinical question: it informs integrated diagnosis, prognosis, and treatment selection (17, 18, 103–105). These considerations contribute to the overall benefit-risk assessment of surgery and patient-centered care, but they do not identify intraoperative functional boundaries or replace patient-specific functional mapping.

Against this backdrop, the importance of ECoG and related neural-interface technologies has grown. ECoG is fundamentally a recording modality with high temporal resolution and spatial coverage determined by electrode placement. It records local cortical activity, including epileptiform discharges, stimulation-induced after-discharges, HFOs, and, in dedicated paradigms, task-related activity (13–16, 46, 47, 60, 61, 63). Passive recording of spontaneous activity must be distinguished from recording-based functional inference and from stimulation-based functional mapping. Task-related ECoG can identify activity associated with a tested function, but this association alone does not establish that the tissue is indispensable for that function (47, 62, 63). When cortical electrodes are used to deliver stimulation and test function, the mapping component is attributable to DES; ECoG records the accompanying activity and may detect after-discharges. An ECoG workflow that links signal acquisition, feature extraction, interpretation, and surgical decisions is more accurately described here as a “brain-signal-based intraoperative decision-support interface.” The term “quasi-BCI,” if used for this workflow, denotes an expanded conceptual analogy adopted in this review, not an established BCI classification. ECoG alone is neither a complete conventional BCI nor an independent method for defining safe resection margins (33–35, 46, 47).

Abnormal peritumoral networks provide complementary information for brain tumor surgery. Existing research suggests that HFOs can be candidate markers of tumor-associated epileptic activity (13, 60, 61); studies of peritumoral hyperexcitability in high-grade gliomas also suggest links to tumor-neural interactions (15). The clinical meaning of HFOs is not uniform: one small retrospective study did not find that HFO resection predicted postoperative seizure freedom (13). Neither HFOs nor hyperexcitability identify a functional boundary. Supratotal-resection studies report possible seizure and oncological benefits in selected patients, but do not validate electrophysiological abnormalities as stand-alone resection limits (14, 106–108). Battista et al. retrospectively analyzed a prospectively enrolled cohort of 15 patients with epileptogenic low-grade gliomas. Intraoperative ECoG identified high-risk epilepsy cortical areas, whereas preoperative navigated transcranial magnetic stimulation (nTMS), checked with intraoperative cortical mapping, provided separate information about functionally eloquent cortex. Resection avoided these functional sites. The ECoG/nTMS-tailored supratotal-resection group had better seizure outcomes than the gross-total-resection group, and no permanent neurological deficits were observed during follow-up (14). These preliminary findings support integrating electrophysiological targets with independently assessed functional constraints; they do not establish equivalence between epileptogenic and functional boundaries, prove absence of neurological risk, or demonstrate an oncological benefit attributable to ECoG.

The complementary role of recording is particularly relevant to preservation of language and higher-order functions. Cortical and subcortical DES remain reference methods for intraoperative functional mapping, supported by outcome evidence from glioma surgery (102). Stimulation can elicit after-discharges or seizures, while task-based mapping requires patient cooperation and samples selected sites and functions (47, 62). High-density arrays and neural decoding may augment this approach; a transition from “stimulate-observe” to “record-infer” as a replacement strategy has not been established. Konrad et al. used a 1,024-electrode array in eight brain tumor patients alongside conventional mapping: somatosensory evoked-potential phase reversal was resolved at 0.4 mm, and speech-related activity was recorded in a region confirmed by direct cortical stimulation (46). This demonstrates recording feasibility, not submillimeter validation of safe resection boundaries. Voskoboynikov et al. investigated automated recording-based speech mapping using machine learning in 14 patients with chronically implanted stereo-EEG electrodes, using stimulation-derived labels as the reference (47), while gamma-modulation visualization provides another example of recording-based functional assessment (63). Such approaches infer task-related involvement and require validation against stimulation mapping and postoperative outcomes before they can independently guide tissue removal (47, 62–64). Flexible cortical arrays also support high-fidelity recording and cortical conformability (109, 110). Cortico-cortical evoked potentials, in contrast, require electrical stimulation and measure evoked network responses; they are not a stimulation-free passive ECoG method (48). The potential contribution of these approaches is additional spatial, temporal, and network information within an established function-guided workflow.

Previous studies have also suggested that intraoperative ECoG can help distinguish electrophysiological patterns in tumor and peritumoral cortex (16). This observation is relevant because the peritumoral region may contain infiltrating tumor cells, participate in functional reorganization, and generate epileptic activity. These processes have different biological meanings and potentially different spatial distributions; their coexistence does not define a single electrophysiologically identifiable “functional-network boundary.” An ECoG difference between regions does not by itself delineate microscopic tumor infiltration, identify indispensable cortex, or establish a safe resection limit. Interpretation therefore requires integration with imaging, tissue-based oncological assessment where available, seizure evaluation, and independent cortical and subcortical functional mapping (14, 16, 62, 102). Contemporary BCI-related recording and decision-support tools add a dynamic electrophysiological layer to long-established function-guided surgery; they do not initiate a new transition from anatomical to functional resection.

## 5 Oncological neuroscience and tumor-neuron-immune interactions: the biological extensions of BCI

One key reason why brain-computer interfaces hold significance beyond the purely technical realm in the context of brain tumors and oncology more broadly is that tumor neuroscience is redefining the relationship between tumors and the host nervous system. Traditional views have largely regarded the nervous system as a passive recipient of tumor compression, infiltration, or treatment-induced damage; however, in recent years, a growing body of research has shown that neural activity itself can not only be altered by tumors but may also actively contribute to tumor growth and invasion through circuit remodeling and regulation of the microenvironment (49–55). Recent cancer-neuroscience reviews and experimental studies have consolidated this concept across brain tumors and peripheral malignancies (66, 67, 69–71).

This understanding is most clearly demonstrated in glioma research. Venkatesh et al. were the first to demonstrate that neuronal activity promotes the growth of high-grade gliomas through the secretion of neuroligin-3 (50), and subsequently suggested that this dependency is potentially amenable to intervention (51). In 2019, two landmark studies further revealed deeper mechanisms: first, brain glioma cells can receive glutamatergic synaptic inputs from neurons (52); second, brain gliomas can integrate into host neural circuits at both the electrical and synaptic levels, forming a growth-promoting positive feedback loop (53). Subsequent studies have further demonstrated that glioma synapses can hijack adaptive plasticity mechanisms, thereby driving the synergistic remodeling of the tumor and brain circuits (54, 55). Related reviews have summarized the central nervous system regulation of diffuse glioma growth and invasion, ranging from single-cell electrophysiology to the circuit level (49). The more recent literature further links glioma-neuron synapses, neuronal activity-dependent signaling, and adaptive plasticity to tumor progression (55, 65–67) and circuit-level vulnerability (111–115).

Together, these studies have driven a key paradigm shift: brain tumors are not merely “tumors growing in the brain”, but can, to a certain extent, be viewed as “tumors embedded within brain circuits and co-evolving with them” (49–55). The scope of this concept is not limited to tumors of the central nervous system. In recent years, the focus of tumor neuroscience research has gradually expanded to include various peripheral solid tumors, particularly gastrointestinal tumors and other peripheral solid tumors characterized by significant nerve infiltration, a prominent burden of pain, and a highly complex microenvironment (71–73, 116–118). In gastrointestinal tumors, evidence includes multilevel interactions among sensory nerves, autonomic nerves, stromal cells, and immune cells (119–125); glutamatergic neuron-cancer pseudo-synapses have also been reported in PDAC (126). Precisely for this reason, tumor-neural-immune interactions hold greater translational appeal in gastrointestinal tumors: they are not only associated with pain and local invasion but may also contribute to immune evasion, treatment resistance, and tumor microenvironment remodeling (119–125). Contemporary reviews highlight nerves as active components of the peripheral tumor microenvironment rather than passive anatomical structures (71–73, 116–118).

Among gastrointestinal cancers, pancreatic ductal adenocarcinoma (PDAC) is currently one of the most representative models in neuroimmunological cancer research. PDAC is characterized by high fibrosis, significant neural infiltration, strong immunosuppression, and intractable pain. Neural remodeling can occur at relatively early stages of the disease, and the interactions among neurons, cancer-associated fibroblasts, and immune cells within the tumor microenvironment are increasingly recognized as a critical component of tumor biology (119, 123–126). Existing studies suggest that neuroactive molecules and autonomic-related signals not only participate in pain transmission but may also contribute to the progression of PDAC by influencing the stromal response, local neurotrophic support, and tumor cell behavior (119–121). Neuronal profiling and neural-invasion studies in PDAC (123, 125), together with functional studies (120, 121, 124, 126), support cancer-associated neuronal reprogramming and interactions with stromal and immune compartments; related mechanisms are reviewed elsewhere (119, 129). Other spatial studies characterize immune and stromal states in pancreatic metastasis or IPMN progression (127, 128), but do not independently demonstrate neuronal causation. Thus, in PDAC, neural signals do not merely convey the experience of pain but may simultaneously participate in the structural remodeling of the tumor microenvironment and tumor-promoting processes (119–121, 123–125). Neural remodeling, stromal responses, and immune suppression can interact, but remain distinct processes whose causal links require direct testing.

Beyond PDAC, spatial profiling of esophageal squamous cell carcinoma has identified an epithelial-fibroblast niche associated with invasion and immune evasion (130). This study provides microenvironmental context, not direct evidence of neural remodeling or perineural invasion. Direct evidence for neuronal reprogramming and a nociceptor-mediated inter-organ immune circuit comes from head and neck cancer studies (131, 132), and should not be presented as gastrointestinal-cancer-specific evidence. In PDAC, single-cell and spatial analysis of neural invasion directly characterizes nerve-associated fibroblast, Schwann-cell, macrophage, and malignant-cell states (125). These findings support cross-tumor comparison while preserving the distinction between spatial association, causal neural mechanisms, and their tissue of origin (**Figure 3**).

**Figure 3:**
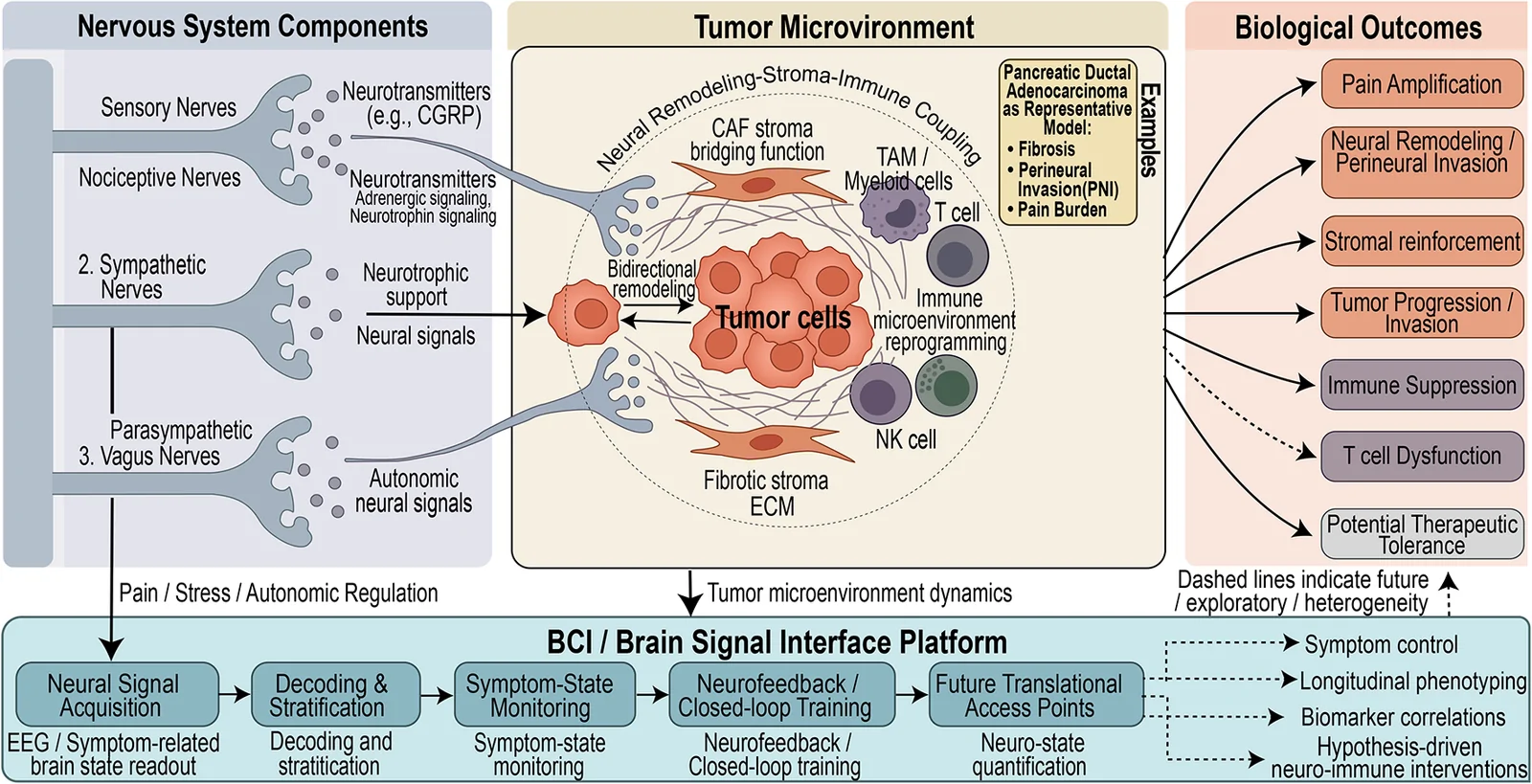
Tumor-nerve-immune interactions in gastrointestinal tumors and potential access points for BCI/brain-signal interfaces. This figure illustrates how sensory, autonomic, and stress-related neural regulatory signals can influence the microenvironment of gastrointestinal tumors through pathways such as neurotransmitters, neuropeptides, and neurotrophic support, and together with cancer-associated fibroblasts, myeloid cells, T cells, and NK cells, form a coupled network of neural remodeling, stromal response, and immune reprogramming. This process may be associated with pain amplification, perineural infiltration, immune suppression, local invasion, and tumor progression. The lower part of the figure illustrates potential access layers for BCI/brain-signal interfaces, including neural state acquisition, decoding layers, symptom monitoring, neural feedback, and future closed-loop translation pathways. It is important to emphasize that current evidence primarily supports the use of BCI for quantifying neural states and monitoring symptoms, while its direct regulation of the tumor immune microenvironment or tumor outcomes remains largely exploratory.

From an immunological perspective, the tumor-nerve-immune interaction warrants attention because the nervous system may not merely be a bystander in tumor immune evasion, but rather an orchestrator and amplifying participant in the process. On the one hand, neurotransmitters, neuropeptides, and neurotrophic factors can act directly or indirectly on immune cells, influencing NK cell cytotoxic activity, macrophage polarization, and T cell functional states (69, 70, 124, 135); on the other hand, chronic pain, persistent stress, and autonomic imbalance may also alter inflammatory responses and immune cell recruitment through neuroendocrine-immune pathways (69, 70, 135). In solid tumors such as gastrointestinal cancers, which are characterized by both a high inflammatory burden and a high stromal barrier, these processes likely collectively shape a microenvironment that both supports tumor infiltration and limits effective antitumor immunity. Therefore, the significance of neuromodulation in this context is not merely a secondary issue related to clinical symptoms, but may touch upon the core mechanisms of tumor biology. Nociceptor-CAF-NK interactions in PDAC (124) and a nociceptor-mediated immune-escape circuit in head and neck cancer (131) provide direct examples of neural regulation. Macrophage-driven T-cell exhaustion (133) and NK-cell therapeutic strategies in pancreatic cancer (134) provide complementary immune context; neither study establishes a neural mechanism by itself.

However, it should be emphasized that the current body of evidence regarding neuroimmune regulation in gastrointestinal tumors still primarily comes from animal models, histological studies, and single-cell/spatial omics analyses; high-level evidence that can directly support clinical interventions remains limited. Therefore, a more prudent statement would be: the regulatory role of the nervous system in the gastrointestinal tumor microenvironment has strong biological plausibility, but whether it can be reliably and controllably translated into clinical treatment strategies remains to be further validated. In particular, there is still a lack of a sufficient and continuous chain of clinical evidence linking the notion that “the nervous system participates in tumor immune regulation” to the conclusion that “neural modulation can improve tumor outcomes”. Therefore, translational claims should remain proportionate: the biological rationale is strong (69–71, 116, 118), but prospective interventional trials linking neuromodulation to immune or survival outcomes are still needed.

It is against this backdrop that the significance of BCIs-or, more broadly, brain-signal interfaces-begins to emerge, though their role must be precisely defined. In the context of brain tumors, BCIs can serve as a platform for recording and decoding peritumoral activity and abnormal synchronization, alongside independent assessment of functional constraints. The utility of brain-signal interfaces in brain tumors should not be viewed merely as a tool to assist in surgery; they also provide a crucial window into tumor-neuron interactions, peritumoral network instability, and the mechanisms underlying disease progression. High-frequency oscillations (HFOs), peritumoral hyperexcitability, and focal synchronous activity are thus no longer merely signals associated with complications but may become the most actionable input variables in closed-loop monitoring and intervention systems (13, 15, 16, 46, 47, 49). For gastrointestinal tumors, the value of BCI is more likely to be realized first in the quantification of neural states and the visualization of neurological symptoms, rather than in direct tumor control. Specifically, cancer pain, visceral pain, chemotherapy-induced peripheral neuropathy, anxiety, depression, and chronic stress may all form complex couplings with tumor-neural-immune interactions. If these neural states can be dynamically monitored and modulated through EEG, neurofeedback, closed-loop training, or related brain-computer interface methods, this holds promise for providing a new platform for supportive therapy and mechanistic research for patients with gastrointestinal tumors (56, 76). The immediate significance of such platforms lies not in directly inhibiting tumor growth, but in transforming previously disparate pain, emotional, stress, and functional states into quantifiable, trackable neural phenotypes that can be correlated with immune or outcome indicators.

For gastrointestinal tumors like pancreatic cancer, which have a particularly high pain burden, the above approach may have greater practical significance. Persistent pain is not only a major damaging factor at the patient’s experience level, but may also have a broader impact on the overall host state through neuroinflammation, central sensitization, and autonomic imbalance (43–45). A randomized, double-blind, placebo-controlled trial reported symptom improvement with targeted and placebo neurofeedback in chronic CIPN, with continued improvement at one month only in the targeted BCI group (56), which at least suggests that brain signal interfaces have practical operability in the management of cancer-related chronic neurological symptoms. Although there is currently no evidence that similar strategies can directly improve the immunotherapy response to PDAC or other gastrointestinal tumors, from the perspective of study design, incorporating neural signal readout, changes in pain status, autonomic indicators, and immunophenotypes into the same prospective framework has clear methodological value (123–126, 129, 131).

Therefore, if we reinterpret BCI from the perspective of gastrointestinal tumors, its more appropriate role is not to “directly treat gastrointestinal tumors via brain-computer interfaces,” but rather to serve as an interface platform linking symptom management, neural state monitoring, and research into the neuroimmune mechanisms of tumors. On one end, it connects to patient-perceived phenotypes such as pain, fatigue, mood, and cognition; on the other end, it connects to autonomic neural activity, neuroinflammation, central nervous system plasticity, and potential changes in the immune microenvironment. This proposed research framing draws on clinical benefit assessment (74), engineering reliability (75), and user-centered usability evaluation (79); these sources do not demonstrate a clinical BCI effect on gastrointestinal tumor biology.

From a broader oncological perspective, tumor neuroscience and tumor-neural-immune interactions suggest that tumors may not merely be tissue lesions, but rather dynamic events deeply embedded within the host’s neural-immune network. In the case of brain tumors, this embedding manifests as direct coupling between neuronal activity, tumor-neuron synapses, and the remodeling of brain circuits (50–54); for gastrointestinal tumors, it manifests more as the synergistic remodeling of neural networks, autonomic signals, stromal responses, and immunosuppressive networks (119–122). The potential value of BCIs and related brain-signal interfaces lies precisely in providing new tools for observing, stratifying, and validating these two distinct levels of “neural embedding.” If tumors can utilize neural activity to reshape the host environment, then neural signal monitoring and closed-loop regulation may also become new entry points for future interventions in tumor-related processes. In this sense, the ultimate potential value of BCIs in oncology may lie not merely in the development of more advanced devices, but in developing a neural-network perspective in oncology that connects symptom management with research into disease mechanisms and microenvironmental regulation.

## 6 Technical bottlenecks, gaps in evidence, and challenges in clinical integration

Although the aforementioned studies have significantly expanded the scope of BCI applications in oncology, their clinical translation still faces multiple constraints. A fundamental reason lies in the fact that, whether it concerns neurofeedback in cancer pain management, peritumoral network recordings in brain tumors, or the extension of mechanisms within the tumor-neural-immune interaction framework, the current evidence remains largely exploratory, heterogeneous, and scattered across different clinical settings (36–38, 68, 74, 75). Particularly for extracranial solid tumors such as gastrointestinal cancers, although significant biological correlations have been demonstrated between neuromodulation and the immune microenvironment (70, 71, 124, 125, 131), there remains a considerable distance to be covered before reproducible and standardized clinical pathways can be established.

First, the conceptual boundaries are not yet entirely clear. Many current studies in oncology concern neural recording interfaces, intraoperative electrophysiology, neurofeedback, or closed-loop neuromodulation; not all meet the definition of a conventional BCI (33–38). Routine ECoG recording and DES mapping are not automatically BCIs. “Quasi-BCI” is used only as an explicitly expanded conceptual interpretation; “brain-signal-based intraoperative decision-support interface” is preferred for the surgical workflows discussed here. Oncological, anatomical/radiological, functional, and epileptogenic/electrophysiological assessments must also remain distinct: electrical abnormality alone does not establish functional expendability or a safe resection boundary. Clinical evaluation should assess real-world benefit and patient-reported outcomes (74), reliability and long-term robustness (75), and usability in terms of effectiveness, efficiency, workload, and satisfaction (79). Longitudinal implanted-BCI studies provide a separate basis for monitoring device- and procedure-related adverse events (81). These sources address different evaluation domains and do not establish oncology-specific effectiveness (**Table 2**).

**Table 2:** Evidence map for major claims and translational readiness.

Domain/major claim	Representative references	Evidence type and level	What the evidence supports	Residual gap
Cancer burden and cancer pain	(1, 4–6, 27–32)	Epidemiology, guidelines, and clinical practice recommendations; Level A.	Cancer burden is high; cancer pain remains prevalent and requires structured multimodal management.	Direct evidence for BCI as standard cancer-pain care is not yet available.
CIPN and neurofeedback-based BCI	(7, 56–59)	Guideline 7; CIPN RCT 56; broader pain reviews 57,58 (A); neuropathic-pain clinical study 59 (B).	CIPN requires symptom-directed management; neurofeedback is a promising adjunct, with a substantial placebo response in the controlled trial.	Larger multicenter sham-controlled trials and mechanistic neural endpoints are needed.
Brain-tumor electrophysiology and surgical mapping	(13–16, 46, 47)	Clinical electrophysiology and early recording/mapping studies; Level B. Ref. 47 uses chronic stereo-EEG, not a glioma-resection cohort.	ECoG and HFOs characterize cortical activity; task-based recordings may support functional inference alongside independent stimulation mapping.	Abnormal activity does not define eloquence, infiltration, or a safe resection limit; prospective outcome validation remains limited.
Glioma-neuron circuit biology	(50–54, 66, 67)	Mechanistic and conceptual cancer-neuroscience studies; Level C/D.	Neuronal activity, glioma-neuron synapses, and circuit integration can support glioma growth and remodeling.	Translation into clinically actionable monitoring or intervention remains exploratory.
Peripheral tumor-neural-immune mechanisms in PDAC and other solid tumors	(70, 71, 123–126, 129, 131)	Mechanistic/translational studies 123-126,131 (C); reviews 70,71,129 (D). Ref. 131 concerns head and neck cancer.	Neural remodeling, nociceptor-CAF-NK interactions, neural invasion, and neuroimmune circuits can shape tumor biology.	Direct clinical evidence that neuromodulation improves immune response or survival is lacking.
Clinical BCI implementation and governance	74,75,79,81-92	Evaluation/engineering reviews 74,75 (D); usability and safety studies 79,81 (B); ethics/legal analyses 82-92 (D).	Benefit, reliability, usability, safety, privacy, consent, agency, and mental integrity must be treated as design constraints.	Regulatory standards, data-governance models, and oncology-specific deployment pathways remain immature.

Evidence level definitions: Level A=guideline, meta-analysis, systematic/scoping review, or randomized trial; Level B=clinical observational, intraoperative, or first-in-human engineering study; Level C=mechanistic, animal, single-cell, spatial, or translational biological study; Level D=expert review, conceptual framework, ethical, legal, or regulatory analysis. These labels describe study design, not a formal certainty-of-evidence hierarchy; relevance to oncology and risk of bias must be assessed separately.

Second, the existing evidence remains highly fragmented. Whether in the context of cancer pain or brain tumors, patient populations exhibit significant heterogeneity, and many direct BCI/neurofeedback and early recording studies remain small-scale and exploratory (46, 47, 56, 57). Larger observational supratotal-resection cohorts (107) and surgical evidence reviews (106) concern resection strategies and should not be counted as BCI efficacy trials. Significant variations across studies in terms of device type, training duration, signal features, feedback strategies, and outcome measures make direct comparison of results difficult and hinder the establishment of standardized protocols. Particularly in the field of cancer pain, although BCI has been supported by randomized controlled trial evidence (56), the overall strength of the evidence remains insufficient to support widespread adoption. For future oncology trials, performance status, fatigue, cognition, bleeding and infection risk, and competing care priorities should therefore be prespecified clinical feasibility considerations.

Third, the clinical utility and accessibility of BCIs still need to be significantly improved. Although invasive systems offer higher accuracy, the surgical risks and long-term maintenance costs must be carefully weighed in cancer patients; non-invasive systems are easier to implement, but they still have significant limitations in terms of signal quality, noise resistance, and individual adaptability (23, 33–35, 75, 81). Furthermore, cancer patients often experience fatigue, cognitive fluctuations, and changes in their condition, factors that further complicate real-world training and deployment. Rapid calibration has been demonstrated as an engineering result (80); patient benefit and usability require separate evaluation (74, 79), while bias and responsible interpretation are governance considerations (85, 89).

Fourth, there is an inherent tension between algorithmic performance and clinical interpretability. Neural signal decoding continues to benefit from artificial intelligence methods (19–21, 39, 42, 80), but high-risk clinical settings are not suitable for relying on completely non-interpretable “black-box” systems. A clinically useful platform should balance decoding performance and calibration burden (80) with robustness (75), patient-relevant benefit (74), and transparent, responsible interpretation (85, 89).

Finally, ethical and regulatory issues must not be underestimated. Recent neuroethics and neurorights scholarship highlights privacy, data ownership, consent, agency, and mental integrity as central issues for neural data governance (82–88). Brain signal data is highly sensitive, neurostimulation may alter subjective experience, and cancer patients are often in a relatively vulnerable state when making decisions. Responsible-development recommendations and analyses of direct-to-consumer neurotechnologies inform consent, data governance, justified efficacy claims, and withdrawal options (89–92); they are ethical guidance rather than oncology-specific clinical validation or a single established regulatory standard.

From an engineering implementation perspective, the next phase of BCI in oncology should prioritize five linked requirements: reliable neural-state biomarkers, rapid and low-burden calibration, interpretable and bias-aware decoding models, seamless integration with surgical and supportive-care workflows, and explicit governance of neural data, consent, and patient agency. These priorities are especially important in oncology, where performance status, treatment-related fatigue, infection risk, end-of-life decision-making, and fluctuating symptom states can undermine device adherence and trial validity. Future platforms should therefore be evaluated as clinical engineering systems rather than isolated algorithms, with endpoints that combine technical robustness, patient-reported benefit, safety, usability, and biological interpretability. Relevant evidence addresses clinical benefit (74), technical robustness (75), usability (79), and longitudinal safety (81); ethical and legal analyses address governance (82–92).

## 7 Conclusions and outlook

Overall, the development of brain-computer interfaces (BCIs) in the field of oncology is still in its formative stages, but its underlying logic is becoming increasingly clear. Early oncology-specific interventional evidence comes from neurofeedback for chronic CIPN (56, 77), while broader cancer-pain applications remain investigational; another clinically relevant area is the integration of brain-signal recordings and peritumoral network assessment with established stimulation-based functional mapping in brain tumors (13–16, 46–48). Research in tumor neuroscience and tumor-neural-immune interactions provides a forward-looking biological context (49–55, 69–73). In surgery, these interfaces add complementary information while oncological extent, functional constraints, and epileptogenic/electrophysiological findings remain distinct. Together, these three areas suggest that the true value of BCIs in oncology lies not in simply transplanting existing neuroengineering technologies, but in fostering a new framework for understanding disease: tumors are not merely tissue lesions, but also network events in brain tumors (13–16, 48, 60, 61) and molecularly stratified neuro-oncological diseases (17, 18, 103–105, 107); they not only alter neural activity and pain-related networks (43–45, 56, 58, 59), but may also be shaped by neural-circuit mechanisms in glioma (49–55) and peripheral tumor-neural-immune mechanisms (70, 71, 124, 125, 131).

In the future, this field may advance along three parallel paths. First, selected applications may progress from open-loop recording to validated closed-loop regulation, enabling brain signals to contribute to symptom management, risk prediction, and network-based interventions. In brain tumor surgery, such development would complement established DES-guided functional preservation, not replace it on the basis of electrophysiological abnormality alone. Second, it will evolve from single-modality neural signal platforms to multi-modal precision integration, combining brain signals with imaging, pathology, molecular characteristics, and artificial intelligence models. Third, the field will expand from perioperative assistive tools to a comprehensive disease management platform, covering preoperative assessment, intraoperative navigation, postoperative monitoring, and long-term rehabilitation. If this progression is realized, the significance of BCI in oncology may extend beyond the development of new devices to represent a new clinical paradigm: tumors will increasingly be understood by the public in terms of how they are embedded within neural networks, the extent of their disruption, and their controllability. Future trials should prioritize multicenter designs and clinically meaningful BCI endpoints (74, 75), sham-controlled neurofeedback where feasible (57–59, 77, 78), standardized neural and patient-reported endpoints (74, 75), explicit neural-data governance (82–88) and responsible neurotechnology oversight (89–92), and prospective linkage with imaging, molecular pathology, immune profiling, and survival outcomes as a proposed validation strategy (**Figure 4**).

**Figure 4:**
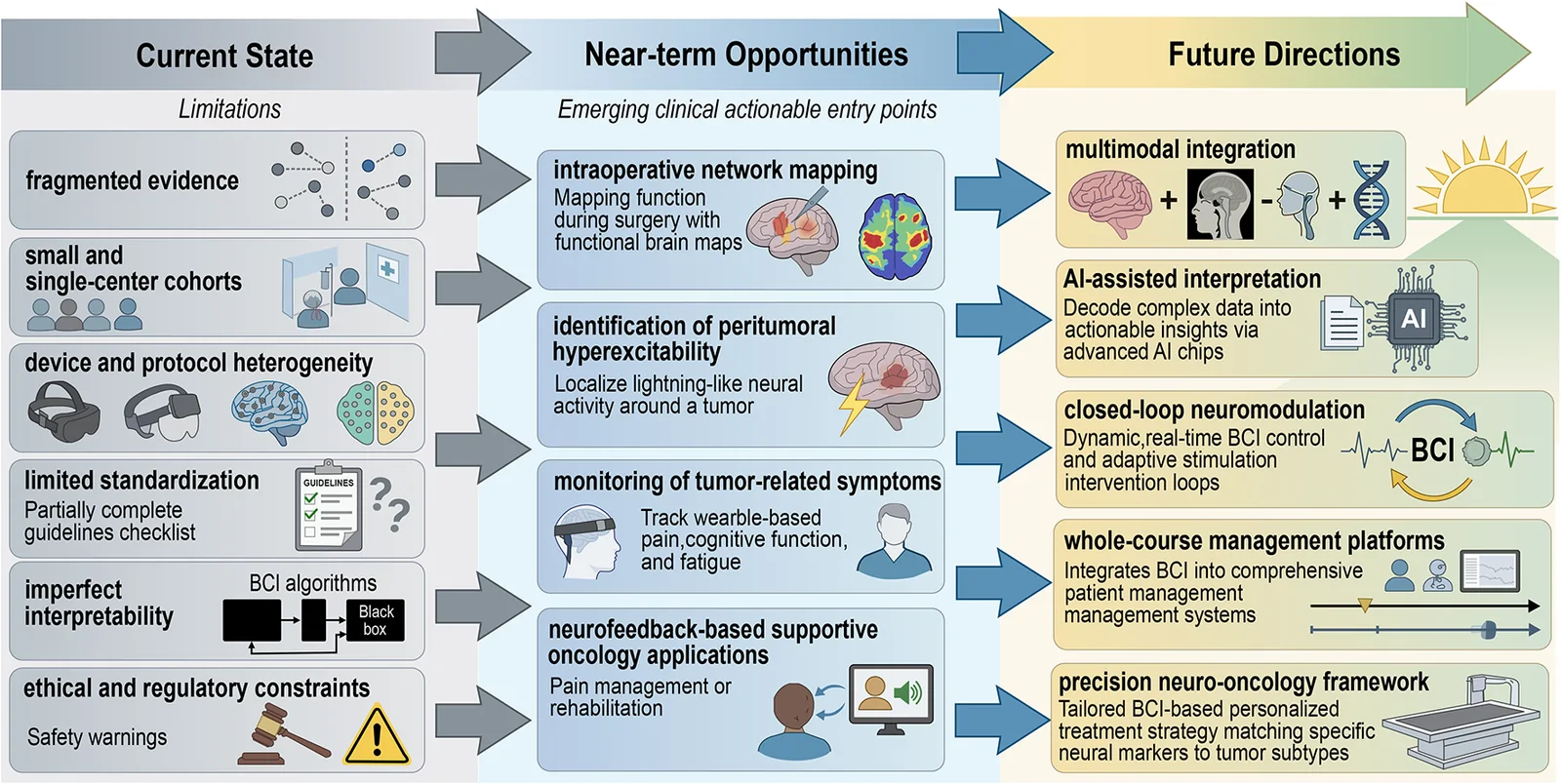
Roadmap for the clinical translation of brain-computer interfaces in oncology. The left panel, headed “Current State” with the subheading “Limitations,” summarizes major limitations, including fragmented evidence, small-sample single-center studies, heterogeneity in devices and protocols, lack of standardization, limited algorithm interpretability, and ethical and regulatory challenges; the middle section highlights clinically feasible entry points in the near term, including recording-based network assessment alongside established DES mapping, assessment of peritumoral hyperexcitability, monitoring of tumor-related symptoms, and neurofeedback applications to support treatment; the right side illustrates future directions, including multimodal integration, AI-assisted interpretation, closed-loop neuromodulation, comprehensive disease management platforms, and a precision neuro-oncology framework. The figure distinguishes emerging recording and decoding tools from established stimulation-based functional mapping; detection of hyperexcitability does not independently identify functional boundaries. The BCI field is evolving from exploratory tools to mechanism-driven clinical platforms, but the prospects for translation still require a foundation of higher-quality evidence.
